# Chondroprotective Effects of Genistein against Osteoarthritis Induced Joint Inflammation

**DOI:** 10.3390/nu11051180

**Published:** 2019-05-27

**Authors:** Feng-Cheng Liu, Chih-Chien Wang, Jeng-Wei Lu, Chian-Her Lee, Shao-Chi Chen, Yi-Jung Ho, Yi-Jen Peng

**Affiliations:** 1 Rheumatology/Immunology and Allergy, Department of Medicine, Tri-Service General Hospital, Department of Orthopedics, Tri-Service General Hospital, Taipei 114, Taiwan; lfc10399@yahoo.com.tw; 2 Department of Orthopedics, Tri-Service General Hospital, Taipei, Department of Orthopedics, Tri-Service General Hospital, Taipei 114, Taiwan; tsghcc@yahoo.com.tw; 3 Department of Biological Sciences, National University of Singapore, Singapore 117543, Singapore; jengweilu@gmail.com; 4 Department of Orthopedics, College of Medicine, School of Medicine, Taipei Medical University, Taipei Medical University Hospital, Taipei 114, Taiwan; chianherlee@yahoo.com.tw; 5 Department of Pathology, Tri-Service General Hospital, National Defense Medical Center, Taipei 114, Taiwan; chort0808@gmail.com; 6 School of Pharmacy, National Defense Medical Center, Taipei 114, Taiwan; ejung330@gmail.com

**Keywords:** osteoarthritis, genistein, IL-1β, inflammation

## Abstract

Genistein is an isoflavone extracted from soybean (Glycine max). This compound has anti-inflammatory, anti-oxidative, and anti-cancer effects; however, the mechanism underlying the effects of genistein on IL-1β-stimulated human osteoarthritis (OA) chondrocytes remains unknown. Our objectives in this study were to explore the anti-inflammatory effects of genistein on IL-1β-stimulated human OA chondrocytes and to investigate the potential mechanisms which underlie them. Our results from an in-vitro model of osteoarthritis indicate that genistein inhibits the IL-1β-induced expression of the catabolic factors nitric oxide synthase 2 (NOS2), cyclooxygenase 2 (COX-2), and matrix metalloproteinases (MMPs). Genistein was shown to stimulate Ho-1 expression, which has been associated with Nrf-2 pathway activation in human chondrocytes. In a rat model, genistein was also shown to attenuate the progression of traumatic osteoarthritis. Taken together, these results demonstrate the effectiveness of genistein in mediating the inflammation associated with joint disorders. Our results also indicate that genistein could potentially serve as an alternative therapeutic treatment for OA.

## 1. Introduction

Osteoarthritis (OA) is the most common form of chronic arthritis. This condition has been linked to excessive weight, injury, and genetic mutations [1,2,3]. OA involves the progressive destruction of the extracellular matrix (ECM) in articular cartilage and bone, which leads to chronic joint pain, particularly among the elderly [4,5,6,7]. Treatment for OA is mainly limited to analgesics and other symptom-focused methods [8,9]. Articular cartilage degradation is caused by (1) biochemical changes related to structural and metabolic deviations and (2) an imbalance between synthesis and degradation pathways [10]. Numerous animal models have been used to study the pathogenesis of OA and the therapeutic efficacy of new treatment modalities [11,12]. The cause of OA is not known; however, it is believed that the pathogenesis of this condition is related to inflammation [13]. Several inflammatory cytokines are involved in pathophysiological processes associated with OA, such as (Interleukin) IL-6, IL-1β, IL-8, and tumor necrosis factor-α (TNF-α) [14,15]. The COX-2/prostaglandin E2 (PGE2) pathway is one of the common pathways to induce inflammation. When the tissue is hurt or becomes infected, the pathway will be activated thought inducing COX-2. Then, the product, PGE2 will be release to induce the inflammation response including heat, pain, redness, and swelling. The response will induce more and more immune cells to target the damaged tissue to help tissue repair and declear the pathogen. It appears that the function of IL-1β is to increase the production of various pro-inflammatory mediators, including PGE2, nitric oxide (NO), disintegrin, and matrix metalloproteinases (MMPs). IL-1β also plays a role in promoting ECM degradation [16]. Therefore, a potential treatment objective for OA in humans could involve inhibiting the inflammation caused by IL-1β [17,18].

Studies have revealed an interaction between members of the Nrf2 and NF-κB pathways [19]. IL-1β-induced nuclear factor-kappaB (NF-κB) plays a role in the production of several inflammatory factors associated with the pathogenesis of OA. This cytokine is also as an important transcription factor involved in the maintenance of cell homeostasis. Nuclear factor erythroid-derived 2-like 2 (NRF2) exerts antioxidant and anti-inflammatory effects via interactions which occur along multiple signaling pathways [20,21]. Nrf2 can be activated through the gene expression of heme oxygenase-1 (HO-1), superoxide dismutase, and NAD(P)H: quinone oxidoreductase-1 [19,22]. However, HO-1 also plays an important role in Nrf2-mediated NF-κB inhibition [19], and activation of the Nrf2 signaling pathway inhibits inflammatory cytokines and NF-κB activation [23,24,25]. Previous in-vivo studies have demonstrated that Nrf2 activation has chondroprotective effects, which may be useful in the treatment of cartilage degeneration [26,27].

In humans and animals, two isoforms of estrogen receptors (ERs), ERa and ERh, are present in chondrocytes [28,29,30,31]. Researchers have demonstrated that cartilage is an estrogen-sensitive tissue in both in-vitro [32,33] and in-vivo [34,35] studies. Genistein is an isoflavone extracted from soybean (Glycine max) that shares structural similarities with selective ER modulators (SERMs), tamoxifen, and the synthetic isoflavone ipriflavone [36]. Tamoxifen and ipriflavone both have beneficial effects on cartilage metabolism and/or the alleviation of OA symptoms [36,37]. Furthermore, flavonoids are also powerful inhibitors of both lipoxygenase and COX-2 activities. Genistein selectively decreases the production of lipopolysaccharide (LPS)-induced COX-2 in human chondrocytes without affecting COX-1 [31].

At present, most of the drugs used in the treatment of degenerative arthritis are non-steroidal anti-inflammatory drugs. However, patients with abnormal liver and kidney function should exercise caution when using these drugs in order to avoid side effects. In the current study, our objective was to determine the efficacy of genistein as an alternative treatment for OA using both in-vitro and in-vivo tests. We also sought to identify the mechanism which underlies the effects of genistein.

## 2. Materials and Methods

### 2.1. Chemicals, Reagents, and Antibodies

Genistein and IL-1β were purchased from Sigma (Sigma, Germany). The antibodies used for Western blotting included anti-MMP-1 (Catalog number: ab52631; Abcam, Cambridge, UK), anti-MMP-2 (Catalog number: ab53015; Abcam), anti-MMP-13 (Catalog number: ab39012; Abcam), anti-Type II Collagen (Catalog number: MAB1330; Millipore Corporation, Burlington, MA, USA), anti-NOS-2 (Catalog number: ab3523; Abcam), anti-Cyclooxygenase-2 (Catalog number: 100109G; Thermo Fisher Scientific, Waltham, MA, USA), anti-Heme oxygenase-1 (Catalog number: ab52947; Abcam), anti-Aggrecan, anti-β-Actin (Catalog number: sc-47778; Santa Cruz Biotechnology, Dallas, TX, USA), anti-rabbit IgG horseradish peroxidase (HRP) (Catalog number: P0217; DAKO, CA, USA), and anti-mouse IgG (HRP) (Catalog number: P0161; DAKO, CA, USA).

### 2.2. Isolation and Culturing of Human Chondrocytes

Human chondrocytes were prepared as described in a previous study [38], and chondrocyte samples were obtained in accordance with protocols approved by the Institutional Review Board (IRB) of the Tri-Service General Hospital. Briefly, chondrocytes were harvested using articular cartilage from patients with osteoarthritis. For this, the cartilage was cut into 0.5 cm^2^ pieces using a sterile scalpel. Cartilage specimens then underwent digestion overnight using collagenase type H and 8× collagenase type II (Sigma, St. Louis, MD, USA, C-8015). Chondrocyte cells were collected using a cell strainer, seeded at a concentration of 58 × 10^5^ cells in a 6 cm dish that contained dulbecco’s modified eagle medium (DMEM)/F12 medium with 10% FBS and antibiotics, and left to culture for 3 to 4 days prior to use.

### 2.3. Cell Viability Assay

Cell viability was assessed using 3-(4,5-dimethylthiazole-2-yl)-2,5-biphenyl tetrazolium bromide (MTT) assays, wherein a total of 1 × 10^4^ cells were seeded in 96-well plates. For this, 50 μL of MTT solution (2 g/L) (AMRESCO, Solon, OH, USA) was added to each well, and cells were incubated at 37 °C for 3 h. Cells were then shocked for 10 min, and absorbance was measured at 570 nm using a microplate reader. All MTT assays were conducted three times, and results were normalized with the control group.

### 2.4. Measurement of Nitric Oxide (NO) Concentrations

NO release was characterized by measuring its stable end product, nitrite, in the supernatant. The Griess reaction was performed using fluid containing 1 × 10^5^ cells collected from 24-well plates, and nitrite concentrations were measured using a spectrophotometer. Briefly, for this, an aliquot (100 μL) of culture supernatant was incubated with 50 μL of 0.1% sulfanilamide in 5% H_3_PO_4_ and 50 μL of 0.1% napthylethylenediamine dihydrochloride (NED) at room temperature for 30 min. Absorbance was subsequently measured at a wavelength of 550 nm using a BioTek Synergy HT microplate reader (BioTek Instruments Winooski, VT, USA) [38].

### 2.5. Western Blotting

Following drug treatment, cells were harvested and lysed using radioimmunoprecipitation assay buffer (RIPA buffer) and protease inhibitor cocktail (Roche Molecular Biochemicals, Germany). Total protein concentrations were determined using the Bradford protein assay (Bio-Rad Laboratories, Hercules, CA, USA). For this, equal amounts of proteins were collected from each sample and then loaded into wells for SDS-PAGE. Proteins were then transferred to polyvinylidene fluoride membranes (Millipore, Billerica, MA, USA) to undergo 5% nonfat milk blocking in PBS-T (phosphate buffered saline with 0.1% Tween-20) for 1 h. Following overnight incubation with the appropriate primary antibodies at 4 °C, the membranes were washed using PBS-T and incubated with horseradish peroxidase-conjugated secondary antibodies. HRP was then detected on the membrane using a LumiFast Plus Chemiluminescence Detection Kit (T-Pro Biotechnology, Taiwan) in accordance with the manufacturer’s protocol. The UVP AutoChemi Image System was used to capture and process images, and band densities in Western blot images were quantified using Image J software (National Institutes of Health, MD, USA).

### 2.6. Gelatin Zymography

Gelatin zymography was performed in accordance with methods described in a previous study [38]. Specifically, the culture supernatant was mixed with 4 μL buffer containing 7.5% SDS, 0.15 M Tris (pH 6.8), and 20% glycerol containing 0.05% bromophenol blue. The supernatant mixture was then analyzed using 10% polyacrylamide gel with copolymerized 0.1% gelatin (Sigma, St. Louis, MI, USA). Following electrophoresis, the gel was washed using renaturing buffer (2.5% Triton X-100) 3 times (30 min per wash) before being added to the developing buffer (1M Tris-HCl; 5M NaCl; 100 mM CaCl_2_; Brij 35) for incubation. Following incubation with the gelatinase buffer overnight at 37 °C, the gel was stained using Coomassie brilliant blue for 30 min. The localization of MMP-2 and MMP-9 was then evaluated using Alpha EaseFC software (Alpha Innotech Corp, San Leandro, CA, USA).

### 2.7. Nuclear Extract Preparation and Electrophoretic Mobility Shift Assay (EMSA)

Nuclear extract preparation and EMSA analysis were conducted, with oligonucleotides containing the Nrf-2 binding sites serving as DNA probes. The EMSA experiment was conducted using the LightShift Chemiluminescent EMSA Kit (Catalog number: 22148; Thermo Fisher Scientific), as detailed in our previous report [38,39].

### 2.8. Transfection of Nrf2 siRNA and Measurement of Reactive Oxygen Species (ROS)

Chondrocytes were transfected with siRNA targeting siGENOME SMARTpool NFE2L2 (Nrf2) using DharmaFECT (Dharmacon, Lafayette, CO, USA) in accordance with the manufacturer’s instructions. Nontargeting siRNA was used as a negative control. Following transfection for 48 h, cells were exposed to genistein or IL-1β for the indicated times. The effects of siRNA on Nrf2 expression were assessed using Western blot analysis and by measuring intracellular ROS levels [40]. For this, we incubated 1 × 10^4^ cells/well overnight at 37 °C under 5% CO_2_ in an incubator. The cells were then exposed to genistein or IL-1β for the indicated times, followed by incubation with 20 μM/mL DCFH-DA (sigma) at 37 °C for 30 min. Following treatment, the cells were washed using PBS, and fluorescence intensity was observed using a microscope (Olympus, Tokyo, Japan). ROS fluorescence intensity was evaluated using Image J software.

### 2.9. Anterior Cruciate Ligament Transection (ACLT) Rat Model

The ACLT rat model employed in this study was based on the protocol outlined in a previous study [12,41]. In this model, 8 week-old rats (*n* = 3 per group) were randomly divided into 3 groups, then the injury and treatment groups of animals underwent ACLT surgery. Standard feeding was initiated in both the control and injury groups one week after surgery. Specifically, the treatment group received standard feeding with oral genistein (40 mg/kg). All experimental animals were sacrificed at 12 weeks after the initial treatment. The protocol employed in the animal study was approved by the institutional animal care and use committee (IACUC) of the National Defense Medical Center, Taiwan (Protocol No. IACUC-17-154, IACUC-17-132). Arthritic scoring was based on scores established by the Osteoarthritis Research Society International (OARSI) [42,43].

### 2.10. Hematoxylin and Eosin (H&E) Staining and Safranin-O Staining

Histological changes were evaluated with Hematoxylin and Eosin (H&E) staining. Changes in proteoglycan content were evaluated using Safranin-O/fast green countered with Weigert’s iron hematoxylin staining [38]. The ratio of glycosaminoglycan (GAG) content to positive staining-section area was performed using commercially available Image J software. A region of positive staining was first drawn around the section to encompass the area and the area was calculated.

### 2.11. Statistical Analysis

GraphPad 5.0 software (GraphPad Software, Inc., La Jolla, CA, USA) and Image J software were used for all data analysis. All data presented in this study were averaged from at least three independent experiments. Quantitative data are expressed as mean ± SD. Comparisons were made using the Student’s *t* test [38]. A *P*-value of <0.05 was adopted to delineate statistically significant differences.

## 3. Results

### 3.1. Genistein Inhibited the Production of NO, Matrix Metalloproteinase-2 (MMP-2), and Matrix Metalloproteinase-9 (MMP-9) in Human OA Chondrocytes

**Figure 1:**
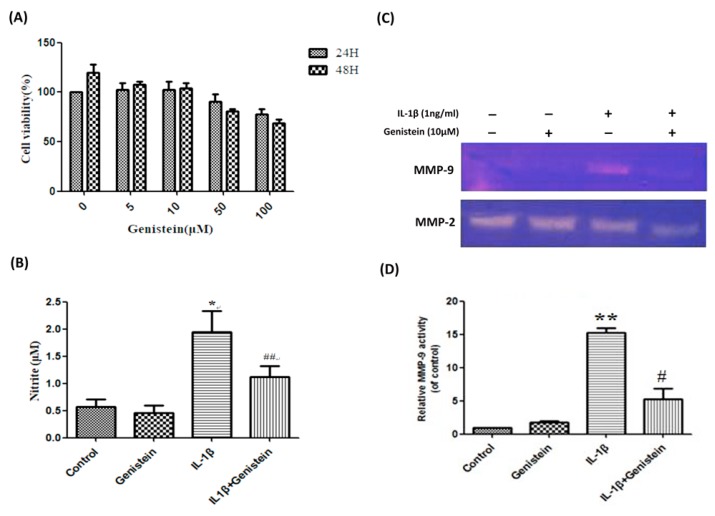
Genistein suppressed IL-1β-induced NO production and enzyme activity while preventing the IL-1β-mediated induction of MMP-9. (**A**) The cytotoxicity of genistein on human chondrocytes was evaluated using MTT assays. For this, chondrocytes were incubated with genistein at various concentrations (0, 5, 10, 50, and 100 μM/mL) for periods of 24 or 48 h. (**B**) Human chondrocytes were stimulated using IL-1β for 24 h prior to treatment with genistein. The production of NO was determined using the Griess reaction. (**C**) The release of MMP-2 and MMP-9 into cell culture supernatants was assessed using gelatin zymography. (**D**) MMP-9 activity was quantified using Alpha EaseFC software. Results are presented as mean ± SD (Obtained from at least three independent experiments). Significance was determined using a *t*-test. Significant differences between the genistein group and the control group are indicated by an asterix (*); significant differences between the genistein group and the IL-1β group are indicated by a pound symbol (#). *, ^#^ *p* < 0.05; **, ^##^ *p* < 0.01.

We first sought to evaluate the effects of genistein on chondrocytes by seeding chondrocytes in 96-well plates (1 × 10^4^ cells/well) with various concentrations of genistein (0, 5, 10, 50, 100 μM/mL). The chondrocyte samples were divided into two groups, which underwent growth for either 24 or 48 h, respectively. We then evaluated cell growth using 3-(4,5-Dimethylthiazol-2-yl)-2,5-diphenyltetrazolium bromide (MTT) assays. Our results revealed that genistein concentrations above 10 μM/mL (i.e., 50 and 100 μM/mL) did not have a statistically significant effect on chondrocyte growth (regardless of the growth duration). We therefore used a chondrocyte concentration of 10 μM/mL in all subsequent experiments (Figure 1A). After stimulating chondrocytes with 1 ng/mL IL-1β and 10 μM/mL genistein for 24 h, supernatant collected from the cell culture was subjected to the Griess reaction to evaluate the effects of genistein on NO release. Following the addition of IL-1β, we observed a significant increase in NO production, compared to the control group (*p* < 0.05). However, the addition of IL-1β in conjunction with 10 μM/mL genistein resulted in a significant decrease in NO production compared with the IL-1β alone group (*p* < 0.01) (Figure 1B).

Gelatin zymography analysis was used to assess the effects of genistein on the extracellular release of MMP-2 and MMP-9. After stimulating chondrocytes with either 10 μM/mL genistein alone or 10 μM/mL genistein in conjunction with 1 ng/mL IL-1β for 24 h, supernatant was collected from the cell culture. We observed a significant increase in MMP-9 levels following treatment with IL-1β alone, compared to the control group, (*p* < 0.01). Conversely, the addition of IL-1β in conjunction with 10 μM/mL genistein resulted in a significant decrease in MMP-9 levels compared with the IL-1β alone group (*p* < 0.05) (Figure 1C,D).

### 3.2. Genistein Reduced Inflammation and Oxidative Stress in Human OA Chondrocytes

**Figure 2:**
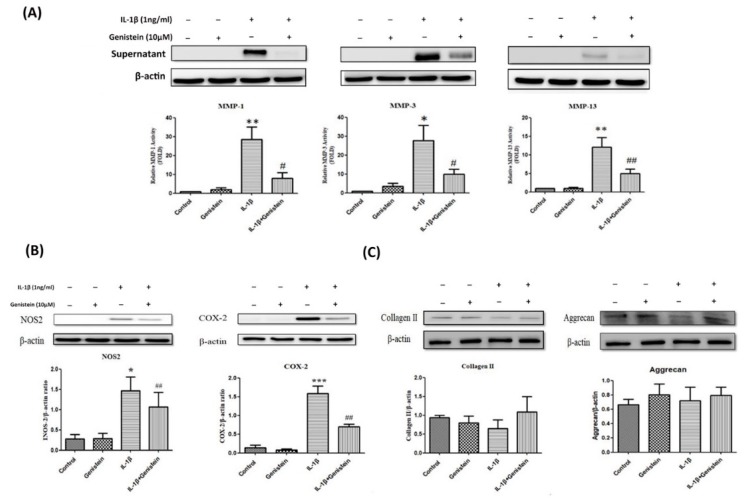
Effects of genistein on protein expression levels of MMP-1, MMP-3, MMP-13, NOS2, COX-2, aggrecan, and collagen II in IL-1β-induced human chondrocytes. (**A–C**) Human chondrocytes were stimulated using IL-1β and then treated with genistein for 24 h. The expression of MMP-1, MMP-3, MMP-13, NOS2, COX-2, aggrecan and collagen II was quantified using Image J software. Results are presented as the mean ± SD from at least three independent experiments. Significance was determined using a t-test. Significant differences between the genistein group and the control group are indicated by an asterix (*); significant differences between the genistein group and the IL-1β group are indicated by a pound symbol (#). *, ^#^ *p* < 0.05; **, ^##^ *p* < 0.01; *** *p* < 0.001.

Western blot analysis was used to evaluate the effects of genistein on the release of MMP-1, MMP-3, and MMP-13 into the extracellular matrix. For this, supernatant was collected from the cell culture after stimulating the chondrocytes with 10 μM/mL genistein and 1 ng/mL IL-1β for 24 h. Following the addition of IL-1β alone, we observed a significant increase in MMP-1, MMP-3, and MMP-13 levels, compared with the control group (*p* < 0.01; *p* < 0.05; *p* < 0.01, respectively). Conversely, treatment with IL-1β in conjunction with 10 μM/mL genistein resulted in a significant decrease in MMP-1, MMP-3, and MMP-13 levels (*p* < 0.05; *p* < 0.05; *p* <0.01, respectively) (Figure 2A). We also evaluated NOS2 oxidative stress and the COX-2 inflammatory index. Under the same experimental conditions, the addition of IL-1β alone resulted in a significant increase in the expression of NOS2 and COX-2 (*p* < 0.05; *p* < 0.001, respectively) compared with the control group, whereas treatment with IL-1β in conjunction with 10 μM/mL genistein resulted in a significant decrease in NOS2 and COX-2 (*p* < 0.001 and *p* < 0.001, respectively) (Figure 2B). Nonetheless, we did not observe significant differences in proteins related to the extracellular matrix, such as aggrecan or collagen II (Figure 2C).

### 3.3. Effects of Genistein on the IL-1β-Induced Nrf2/HO-1 Pathway in Human OA Chondrocytes

**Figure 3:**
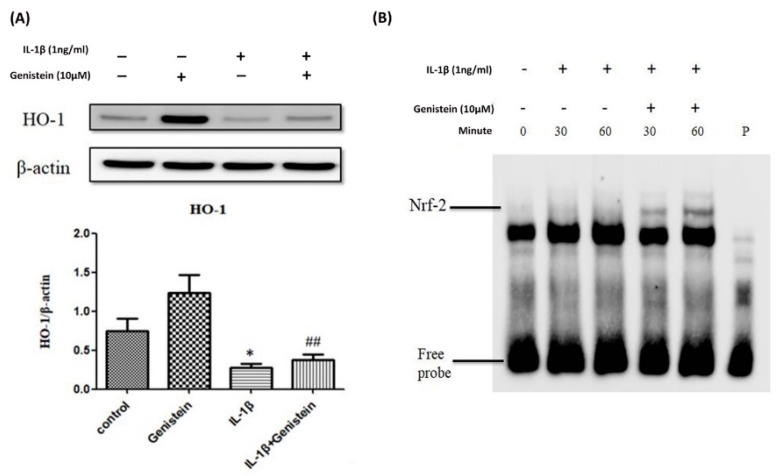
Genistein affected IL-1β-stimulated Nrf2/HO-1 signaling pathways in chondrocytes. (**A**) Human chondrocytes were stimulated using IL-1β and then treated with genistein for 24 h. HO-1 protein expression was quantified using Image J software. (**B**) Nuclear extracts of chondrocytes were treated with IL-1β in the presence of solvent or various doses of genistein for 24 h before the DNA-binding activity of Nrf-2 was analyzed using EMSA. P: probe only; Results are presented as the mean ± SD from no more than three independent experiments. Significance was determined using a *t*-test. Significant differences between the genistein group and the control group are indicated by an asterix (*); significant differences between the genistein group and the IL-1β group are indicated by a pound symbol (#). * *p* < 0.05; ^##^ *p* < 0.01.

We also evaluated the effects of genistein on the HO-1 pathway (downstream from the translation factor Nrf-2) in chondrocytes. We found that pre-treatment with 1ng/mL IL-1β for 24 h significantly reduced HO-1 expression in chondrocytes (*p* < 0.05) compared to the control group, whereas co-treatment with IL-1β and 10 μM/mL genistein significantly increased HO-1 expression (*p* < 0.001). Our results revealed that genistein can significantly recover the IL-1β-induced decreases in Nrf2/HO1 (Figure 3A). We also found that genistein is highly effective in promoting IL-1β-induced Nrf-2 DNA-binding activity in chondrocytes at 30 and 60 min (Figure 3B).

### 3.4. Nrf2 siRNA Inhibits HO-1 Expression and Induces ROS Generation in IL-1β-Induced OA Chondrocytes

**Figure 4:**
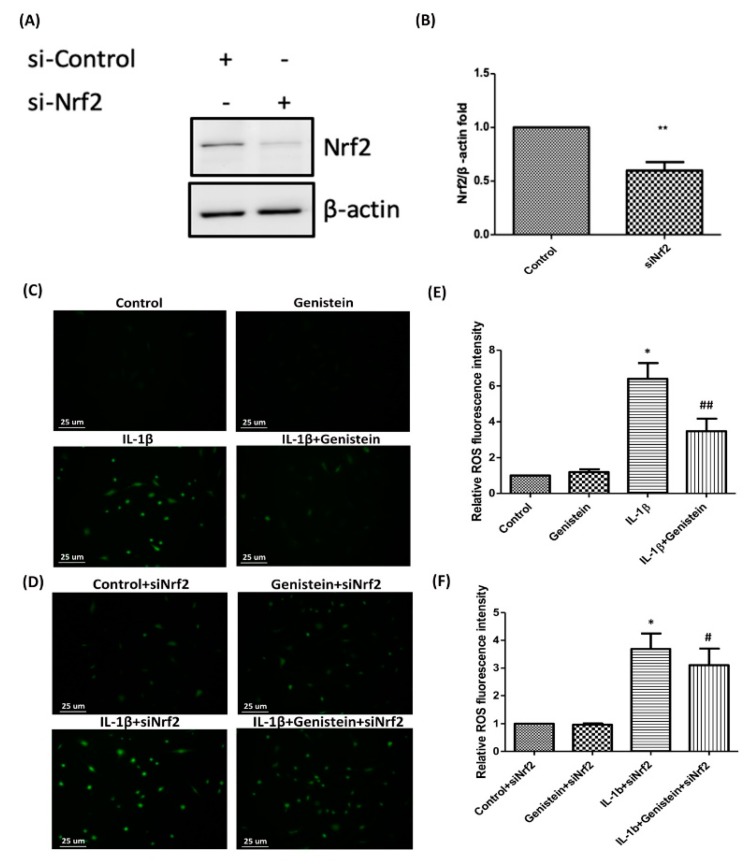
Nrf2 siRNA inhibits HO-1 expression and induces ROS generation in IL-1β-induced human OA chondrocytes. Chondrocytes were transfected with Nrf2-siRNA, IL-1β+Nrf2-siRNA, genistein+Nrf2-siRNA, or IL-1β+genistein+Nrf2-siRNA for 24 h. (**A–B**) Levels of Nrf2 knock-down were detected using Western blot analysis. (**C–D**) Following treatment with genistein for 24 h, control and Nrf2 knock-down cells were stimulated using IL-1β for 24 h. ROS fluorescence intensity was assessed using a fluorescence microscope. (**E–F**) Quantitative analysis of ROS fluorescence intensity was performed using Image J software. Results are presented as mean ± SD from at least three independent experiments. Significance was determined using a *t*-test. Significant differences between the genistein group and the control group are indicated by an asterix (*); significant differences between the genistein group and the IL-1β group are indicated by a pound symbol (#). *, ^#^ *p* < 0.05; **, ^##^ *p* < 0.01. Scale bars = 25 um.

We further confirmed the roles played by Nrf2 and its downstream target enzymes in IL-1β-induced OA chondrocytes. For this, chondrocytes were transfected with si-Nrf2 or an siRNA control. We observed a significant decrease in the expression of Nrf2 in cells transfected with si-Nrf2, compared to the cells transfected with siRNA (*p* < 0.01) (Figure 4A,B). These results also show that knockdown of Nrf2 could affect the genistein-modulated inhibition of IL-1β-induced ROS production was found to be reversed by Nrf2 siRNA (*p* < 0.05) (Figure 4C–F).

### 3.5. Genistein Slowed the Disease Progression of OA in an ACLT Rat Model

**Figure 5:**
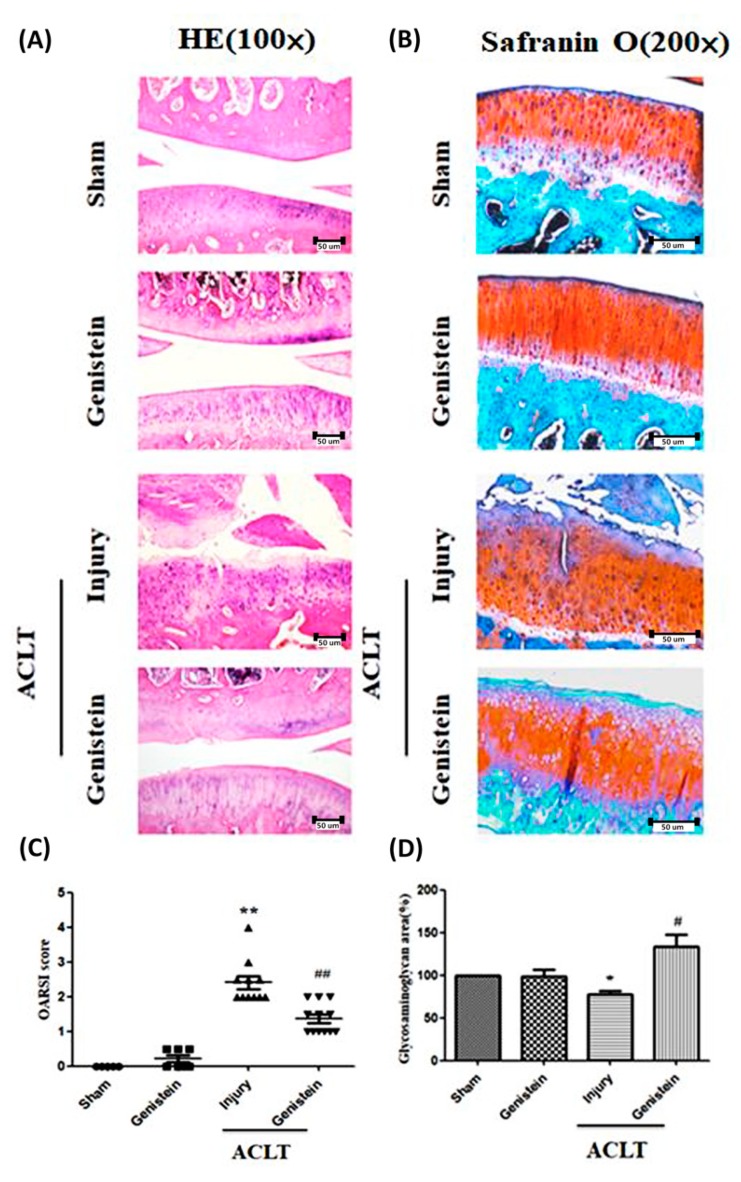
Genistein prevents OA disease progression in a rat model. (**A**) Representative joint sections were obtained from each group of rats at 3 months after treatment with genistein (*n* = 3 for each group). H&E staining revealed signs of inflammation (100×), (**B**) and safranin O staining revealed a reduction in GAG (200×). (**C**) Inflammation and cartilage damage were assessed by assigning OARSI scores to H&E staining results. (**D**) GAG loss status was based on safranin O staining results obtained using Image J software. Significance was determined using a *t*-test. Significant differences between the genistein group and the sham control group are indicated using an asterix (*); significant differences between the genistein group and the ACLT injury group are indicated using a pound sign (^#^ ). *, ^#^ *p* < 0.05; **, ^##^ *p* < 0.01. Scale bars = 50 um.

Our in-vitro results revealed that genistein inhibited IL-1β-induced signaling, thereby preventing the degradation of cartilage matrix and inhibiting inflammation. The anti-inflammatory activity of genistein was evaluated in vivo using an anterior cruciate ligament transection (ACLT) rat model. For this, 8-week-old rats underwent ACLT surgery, which involved cutting and suturing the anterior cruciate ligament. After three months of treatment, articular cartilage in the sham group appeared to be severely worn. Conversely, articular cartilage in the group treated with genistein (40 mg/kg) appeared smooth (Figure 5A). Moreover, OARSI scores of animals treated with genistein were significantly lower than those of the ACLT injury group (*p* < 0.01) (Figure 5C). Safranin O staining further revealed the loss of GAG, wherein the sham group presented lower GAG levels than did the injury group (*p* < 0.05). Finally, we observed that GAG levels in the genistein group were significantly higher than in the ACLT Injury group (Figure 5B). Specifically, GAG positive staining in the ACLT genistein group covered a significantly larger area than in the ACLT Injury group (*p* < 0.05) (Figure 5D).

## 4. Discussion

Age, trauma, and obesity are the main risk factors for cartilage degeneration in OA. The main process involved in degenerative OA is the production of inflammatory factors and oxidative stress factors by cells in joint cavities. Nonetheless, inflammatory cytokines IL-1β and MMPs also play key roles in major joint diseases [44]. Genistein is a major anti-tumor component of isoflavone that has been shown to interact with the ER [45] and reduce the incidence of various hormone-related tumors [46,47]. Genistein derivatives are becoming accepted as novel selective SERMs, particularly when treating issues involving the vascular system, bone, and uterus [48]. It is possible that the estrogen-like structure of genistein enables it to bind to estrogen receptors. Estrogen-like hormones have been shown to prevent and/or ameliorate rheumatoid arthritis (RA) [49]. It is also possible that isoflavones affect cartilage metabolism directly. Estrogen α and β receptors are present in the cartilage of human joints, which indicates that cartilage is also a target of estrogen receptor modulators. Another estrogen-like isoflavone effectively treats OA through estrogen receptor binding to interfere with local estrogen function [49]. Previous researchers have reported that (1) soy isoflavones also inhibit NO production and (2) genistein can down-regulate NO synthase in chondrocytes through the inhibition of tyrosine kinase [50,51]. Indeed, genistein has been shown to inhibit the production of pro-inflammatory molecules, such as COX-2 and NO, in LPS-induced chondrocytes [31]. Our results further revealed that genistein inhibits IL-1β-induced pro-inflammatory molecules, such as the production of NO or NOS2 and COX-2 expression in OA chondrocytes (Figure 1B and Figure 2B).

It is widely known that the NF-κB signaling pathway regulates inflammatory mediators involved in the development of OA [21]. However, genistein inhibits the protein level of COX-2, which is associated with several discrete signaling pathways and the genesis of COX-2 synthesis such as COX-2 is partly controlled by nuclear factor kappa B (NF-kB) [52,53]. COX is a key pro-inflammatory enzyme that converts arachidonic acid into prostaglandins. Inhibition of NF-kB activation is associated with the down-regulation of COX-2 expression and synthesis. Prostaglandins are associated with pain and inflammation in OA; however, this evidence is insufficient to explain the joint inflammation and other symptoms of OA [54].

The up-regulation of IL-1β has been observed in the synovial fluid and cartilage of patients with OA [55]. Pro-inflammatory cytokines, such as IL-1β and tumor necrosis factor α (TNF-α), are believed to cause cartilage damage by inducing the expression of MMPs in chondrocytes. The subsequent synthesis and release of MMPs can lead to matrix breakdown [56,57,58]. Our results in this study revealed that genistein suppresses the IL-1β-induced expression of inflammatory molecules, such as MMP-1, MMP-2, MMP-3, and MMP-13, in OA chondrocytes (Figure 1C and Figure 2A). Previous research has revealed that, in endothelial cells in the human umbilical vein, genistein acts via the NF-kB pathway to protect against inflammation induced by oxidized low-density lipoproteins [59]. The inhibitory effects of genistein on the osteoclast formation of receptor activator of nuclear factor-κB ligand-stimulated osteoclast differentiation can probably be attributed to (1) disruption of the mitochondrial electron transport system and (2) the scavenging of reactive oxygen species through the Nrf2-mediated induction of HO-1 (a phase II antioxidant enzyme) [60]. Our results revealed that genistein may enhance the ability of Nrf-2 to migrate into the nucleus by increasing HO-1 expression, thereby protecting chondrocytes from oxidative stress (Figure 3A,B). Interestingly, the small drug molecule Wogonin plays a similar role in protecting OA chondrocytes [61]. These findings indicate that there may be other similar mechanisms which underlie the protective effects of genistein that still require further clarification.

In the current study, results from a rat model (in which the Th1-predominant immune response was modulated) indicated that genistein (administered through subplantar injection at the site of rheumatoid arthritis (CIA) modulated immune responses in collagen [62]. Genistein was also shown to suppress the expression of IL-1β, IL-6, and TNF-α in the serum, which indicates that it could have the potential to improve symptoms of rheumatoid arthritis in rats [63]. Genistein was also shown to reduce inflammation, joint adhesion, and structural destruction while simultaneously inhibiting vascular endothelial growth factor (VEGF) expression and blocking angiogenesis in synovial tissue [64]. In our ACLT rat model, cartilage surfaces appeared severely worn three months after surgery, which is characteristic of OA. Conversely, in animals that received genistein treatment starting 1 week after surgery, cartilage surfaces were only slightly worn (Figure 5A,C) and GAG was well preserved (Figure 5B,D). We further observed the expression of estrogen receptor β in mandibular cartilage of female rats who received genistein for six weeks. We also observed a decrease in collagen type II and aggrecan in the extracellular matrix of these rats [65]. Nonetheless, we did not observe the same results in intra-articular hyaline cartilage (Figure 2C).

In conclusion, our findings revealed that genistein inhibits the expression of IL-1β-induced inflammatory mediators by targeting the Nrf2/HO-1 pathway in human OA chondrocytes. Genistein reduced the expression of catabolic factors NOS2, COX-2, and MMPs while simultaneously stimulating Ho-1 expression. These effects were in turn shown to be associated with the activation of the Nrf-2 pathway following the administration of IL-1β in an osteoarthritic in-vitro model. Moreover, treatment with genistein was shown to decrease OARSI scores and attenuate the progression of traumatic osteoarthritis in our rat OA model. These results suggest that genistein could serve as an alternative anti-inflammatory agent in the treatment of OA. However, further research will be required to better elucidate the underlying mechanism and clinical efficacy of genistein in treating this condition.
